# Atomic Pairwise Distribution Function Analysis of the Amorphous Phase Prepared by Different Manufacturing Routes

**DOI:** 10.3390/pharmaceutics4010093

**Published:** 2012-01-31

**Authors:** Johan P. Boetker, Vishal Koradia, Thomas Rades, Jukka Rantanen, Marja Savolainen

**Affiliations:** 1 Faculty of Pharmaceutical Sciences, University of Copenhagen, Universitetsparken 2, 2100 Copenhagen, Denmark; Email: jpb@farma.ku.dk (J.P.B.); msa@farma.ku.dk (M.S.); 2 School of Pharmacy, University of Otago, 18 Frederick Street, Dunedin 9054, New Zealand; Email: thomas.rades@otago.ac.nz

**Keywords:** atomic pair-wise distribution functions, Raman spectroscopy, XRPD, amlodipinebesilate

## Abstract

Amlodipine besilate, a calcium channel antagonist, exists in several solid forms. Processing of anhydrate and dihydrate forms of this drug may lead to solid state changes, and is therefore the focus of this study. Milling was performed for the anhydrate form, whereas the dihydrate form was subjected to quench cooling thereby creating an amorphous form of the drug from both starting materials. The milled and quench cooled samples were, together with the crystalline starting materials, analyzed with X-ray powder diffraction (XRPD), Raman spectroscopy and atomic pair-wise distribution function (PDF) analysis of the XRPD pattern. When compared to XRPD and Raman spectroscopy, the PDF analysis was superior in displaying the difference between the amorphous samples prepared by milling and quench cooling approaches of the two starting materials.

## 1. Introduction

The amorphous state of solid compounds is characterized by the absence of orientational and positional long range order, typical for the crystalline solid state [1]. Amorphous forms may however contain some residual order from their crystalline counterparts as well as near order over the length scale of a few molecules [2]. Amorphous forms of drugs are attractive in drug development, especially for poorly water soluble compounds (BCS Class 2 compounds), as they possess a faster dissolution rate and higher apparent solubility than their respective crystalline forms [3]. This advantage, however, goes hand in hand with an increased physical instability, and amorphous systems have a tendency to convert back to the thermodynamically more stable crystalline state (either a metastable or the stable polymorphic form) [4]. Although conventional X-ray powder diffraction (XRPD) is routinely used to analyze non-crystalline material it has to be taken into account that this technique is directly measuring a crystalline property (long range order, leading to diffraction peaks in the diffractograms), the absence of which is in turn interpreted as amorphousness [1]. In contrast, the application of atomic pair-wise distribution functions (PDF) to the diffractograms takes both, diffracted and diffuse X-ray scattering into account, and thus also probes the non-crystalline signals of the diffractograms [2,5,6]. 

The PDF technique utilizes a Fourier transformation of the XRPD diffractograms to produce a trace in a coordinate system. The y-axis in the PDF trace is corresponding to the probability of finding two atoms separated by a distance stipulated by the x-axis. The PDF hence assesses the inter-atomic distances of the material [2,7,8,9]. The accuracy of this assessment of inter-atomic distances is directly proportional to the energy of the utilized radiation source [10,11]. It is therefore inferred that using low energy X-rays, as is done in this study, does not provide an assessment of the atomic structure but may nevertheless be sufficient to display changes in the diffuse scattering of the amorphous samples. PDF analysis has been employed for many decades within the field of material science [12,13]. PDF analysis has more recently been applied within the pharmaceutical sciences on chemical compounds such as piroxicam [8], saquinavir [14], indomethacin [2,5] and polymer-drug mixtures [15,16].

The aim of this study was to investigate the influence of milling and quench cooling on two different forms of amlodipine besilate using XRPD, Raman spectroscopy (as a molecular level technique, commonly used complementary to XRPD) and PDF analysis of the XRPD patterns. 

## 2. Experimental Section

### 2.1. Materials

Amlodipine besilate (AMB) was used as a model active pharmaceutical ingredient (API). The anhydrate form of AMB (AH) was obtained from Matrix Laboratories Limited (Secuderabad, India, batch no. ADP0140208, EP/USP grade). 

### 2.2. Preparation of Dihydrate and Amorphous Forms

The dihydrate form (DH) was prepared from an aqueous slurry by stirring the AH crystals in water for 2 days at 25 °C. The crystals were collected by filtration and dried at 40 °C [17]. Amorphous AMB (AM) forms were prepared by the following methods: quench cooling of DH by heating DH to 106 °C and after dehydration cooling the liquefied sample rapidly in a freezer. Milling of AH was performed in a ball mill (Retsch MM 400, Germany) with two 25 mL jars containing 400 mg of AH and two 9 mm steel beads. Milling time was 60 min at a milling frequency of 25 Hz. 

### 2.3. Raman Spectroscopy

The two solid forms as well as the changes in the solid samples after processing were analyzed using a Raman spectrometer (Control Development Inc., South Bend, IN, USA) equipped with a thermoelectrically cooled CCD detector and a fiber optic probe (InPhotonics, Norwood, MA, USA). The measurements were carried out at room temperature using a 500 mW laser source with a wavelength of 785 nm (Starbright 785S, Torsana Laser Technologies, Skodsborg, Denmark). The integration time was 3 s and each spectrum was an average of 8 scans.

### 2.4. XRPD

X-ray powder diffractograms were measured on a PANalytical X’Pert Pro *θ/θ* diffractometer equipped with a PIXcel detector (PANalytical B.V., Almelo, The Netherlands). A continuous 2θ scan was performed in a range of 2° to 40° using Cu*K*_α_ radiation (λ = 1.5406 Å) with as step size of 0.0390 °2*θ*. The *K*_β_ radiation was eliminated by a nickel filter. The voltage and current applied were 45 kV and 40 mA respectively. The film sample was placed in a 0.2 mm deep sample holder and the measurement was performed at ambient conditions. Data were collected using X’Pert data collector version 2.2 and were analyzed with X’Pert highscore plus version 2.2.4 (both from PANanalytical B.V., Almelo, The Netherlands). 

### 2.5. PDF

PDF analysis by Fourier transformation of the XRPD diffractograms collected from 5° < 2*θ* < 40° was performed using the freeware program RAD developed by V. Petkov. The program setup has previously been described [18]. The program can be obtained from this site [19]. 

### 2.6. Multivariate Data Analysis (MVDA)

MVDA on Raman spectroscopic data and XRPD data before and after PDF analysis was performed using principal component analysis (PCA). Before PCA, standard normal variate (SNV) transformation was performed to remove intensity differences unrelated to the sample composition and the data were then mean centered. PCA, preprocessing and scaling were performed using PLS toolbox version 6.2.1 (Eigenvector Research Inc., Wenatchee, WA, USA).

## 3. Results and Discussion

**Figure 1:**
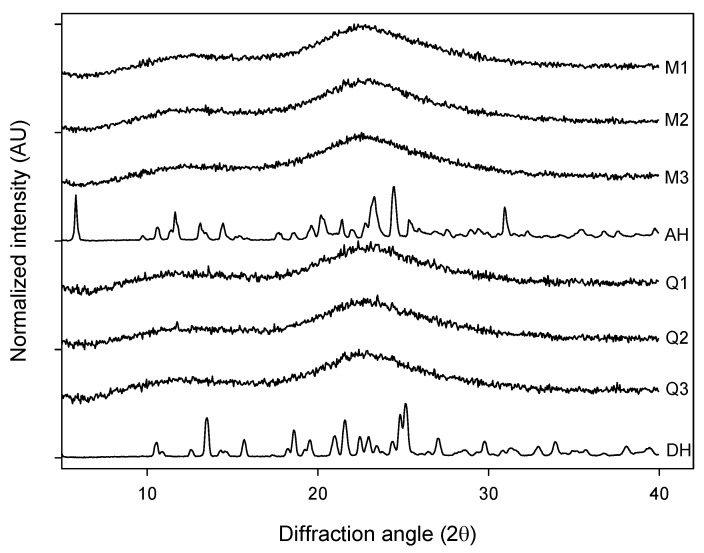
XRPD diffractograms of: milled samples (M1, M2 and M3), quench cooled samples (Q1, Q2 and Q3), crystalline anhydrate (AH) and crystalline dihydrate (DH).

The different forms of AMB were subjected to X-ray powder diffraction measurement. The milled samples of AH (M1, M2 and M3) as well as the quench cooled samples of DH (Q1, Q2 and Q3) displayed XRPD diffractograms that can be characterized as broad and featureless whereas the crystalline anhydrate (AH) and dihydrate (DH) showed patterns similar to the published ones [17,20] (Figure 1). The broad features seen for the processed samples did not provide any visual evidence for differences in the degree of disorder between the milled and quench cooled samples. 

**Figure 2:**
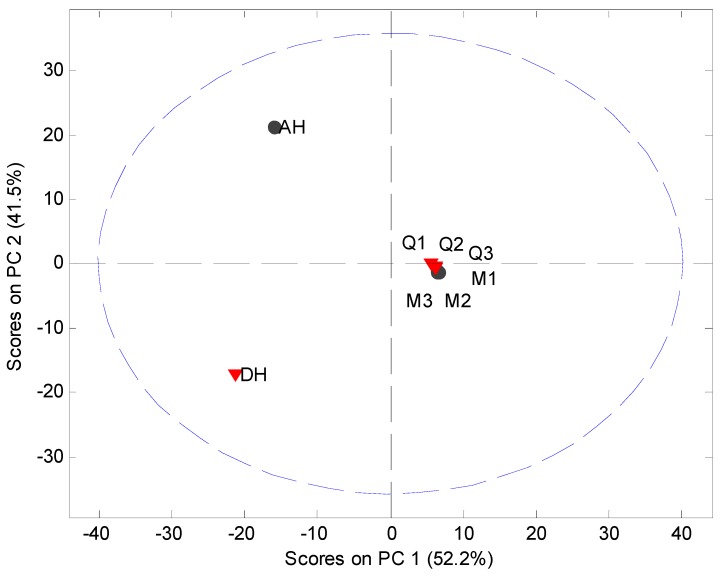
PCA score plot of the original XRPD data (5° 2*θ* to 40° 2*θ*) of milled samples (M1, M2 and M3), quench cooled samples (Q1, Q2 and Q3), crystalline anhydrate (AH) and crystalline dihydrate (DH).

MVDA has been widely used as a fast approach for evaluation of solid state phenomena [21] and is subsequently employed in this study. MVDA was performed using standard normal variate (SNV) correction and mean centering of the 5° to 40° 2*θ* part of the diffractogram (Figure 2). The model explained 93.7% of the variation in the data set using two principal components (PCs). The scores plot indicated that only the crystalline counterparts can be readily distinguished from each other and the processed samples. However, such information is also easily obtained from the diffractograms themselves. Several other models using different preprocessing, multiple PCs and exclusion of the crystalline samples from the data set were tried. However, it was not possible to obtain a separation of the XRPD diffractograms into clusters of the milled and quench cooled samples. 

**Figure 3:**
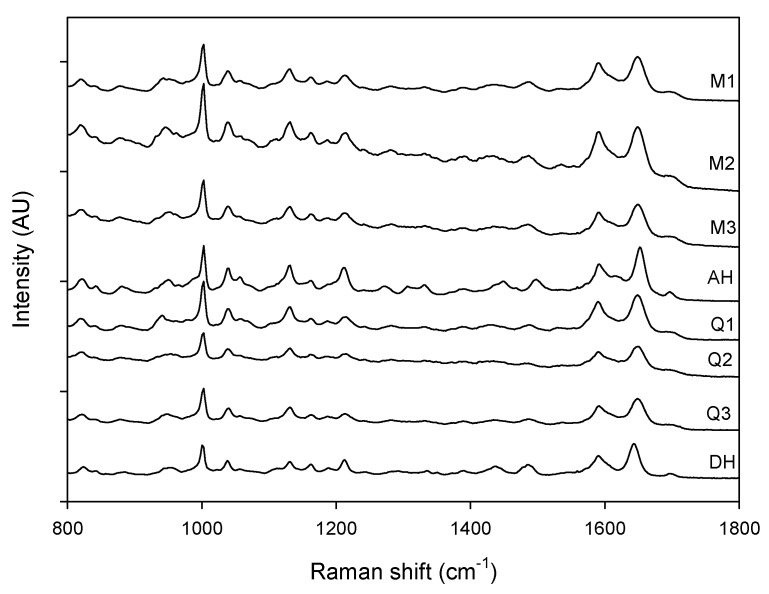
Raman spectra of milled samples (M1, M2 and M3), quench cooled samples (Q1, Q2 and Q3), crystalline anhydrate (AH) and crystalline dihydrate (DH).

**Figure 4:**
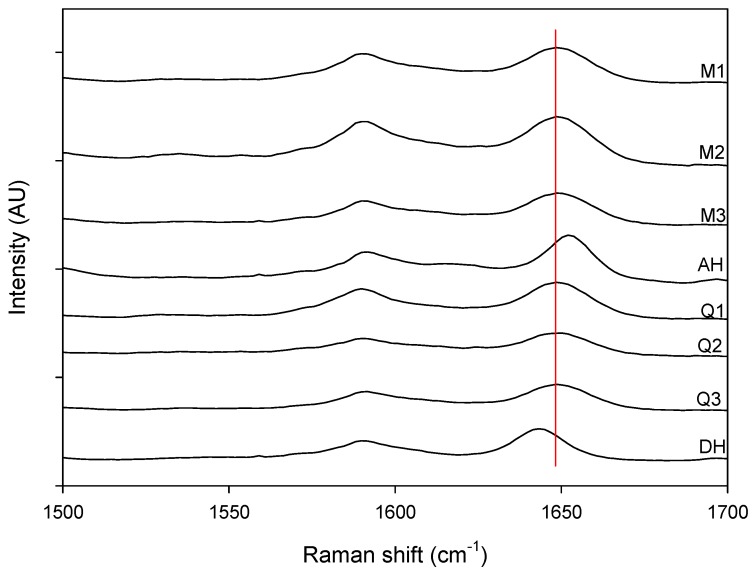
Magnification of the 1500 cm^−1^ to 1700 cm^−1^ spectral region. Milled samples (M1, M2 and M3), quench cooled samples (Q1, Q2 and Q3), crystalline anhydrate (AH) and crystalline dihydrate (DH).

Figure 3 shows that a change could be observed in the Raman spectra for the milled and quench cooled samples. For example a peak shift from 1643 cm^−1^ to 1648 cm^−1 ^occurred during dehydration and a peak shift from 1652 cm^−1^ to 1648 cm^−1^ occurred during milling. This peak at 1648 cm^−1^ (red line in Figure 4) corresponds to the amorphous form and can be used to monitor the solid state transformation [17]. 

**Figure 5:**
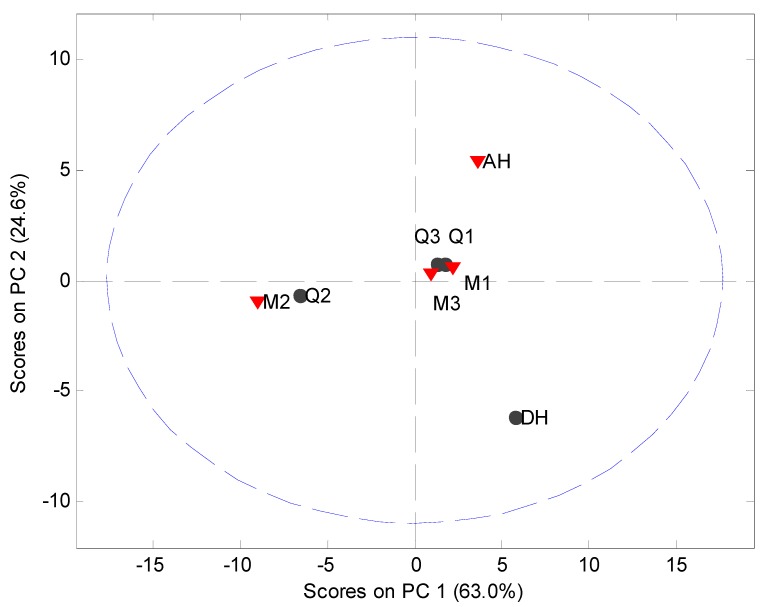
PCA score plot of Raman data (900 cm^−1^ to 1800 cm^−1^). Milled samples (M1, M2 and M3), quench cooled samples (Q1, Q2 and Q3), crystalline anhydrate (AH) and crystalline dihydrate (DH).

MVDA, using SNV correction and mean centering, was utilized on the 900 cm^−1^ to 1800 cm^−1^ spectral region. Four PCs, to explain 97.0% of the variation, were utilized to elucidate differences in the Raman spectra of the processed samples. It was observed that PC 2 is capable of separating the crystalline counterparts. However, no differences could be extracted from the processed samples using the two first principal components because even though two separate groups were formed, each group contained samples from both the milled and quench cooled samples (Figure 5). After inspection of the loadings on PC 1 and the raw data it was apparent that this clustering for PC 1 was caused by intensity differences in the Raman spectra. These intensity differences are most likely related to different distances between the Raman probe and the various samples. The differences in the remaining PCs were also examined, however, these PCs were impaired by a large presence of background noise in the loadings and no useful information was subsequently obtained from them. Finally, MVDA was also performed on the Raman data range from 200 cm^−1^ to 1800 cm^−1^ but this model was also incapable of separating the milled and quenches cooled samples from one another. 

**Figure 6:**
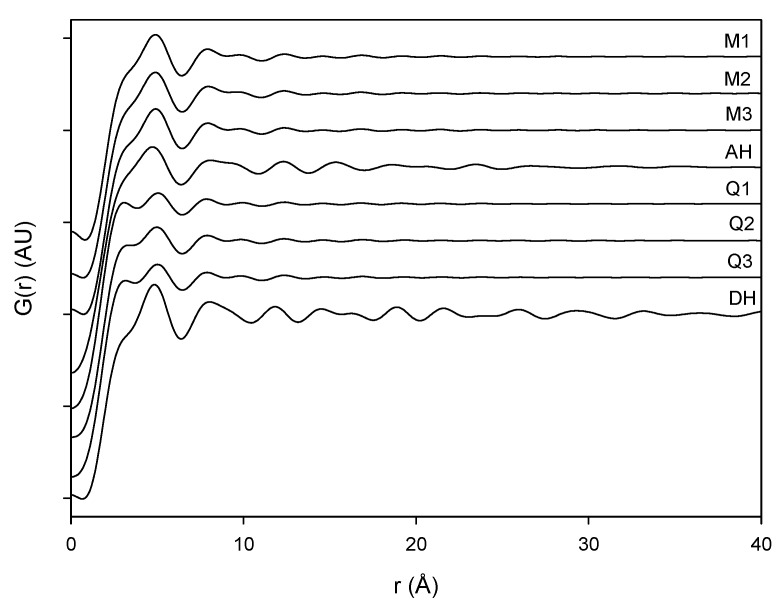
PDF pattern of the milled samples (M1, M2 and M3), quench cooled samples (Q1, Q2 and Q3), crystalline anhydrate (AH) and crystalline dihydrate (DH).

**Figure 7:**
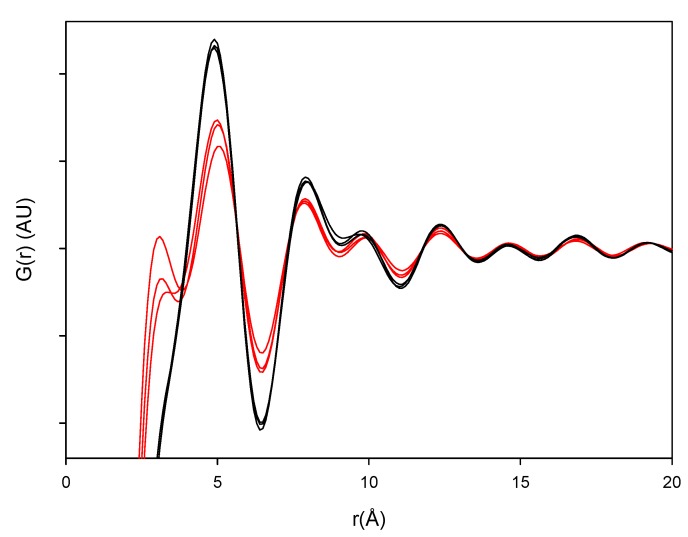
PDF patterns of the processed samples. Milled samples: black lines and quench cooled samples: red lines.

The PDF traces of XRPD data displayed in Figure 6 represent the various milled and dehydrated samples as well as the crystalline DH and AH starting material. It is observed that the PDF trace of all the samples attenuates with varying rates towards unity at distances far away from the origin. Furthermore, it is observed that the crystalline AH and DH starting materials have a peak attenuation that is much lower than the corresponding attenuation for the processed samples. This low PDF trace attenuation of the crystalline samples is a direct consequence of the more ordered structure of these samples compared to their amorphous counterparts. When overlaying the milled samples (black lines) and quench cooled samples (red lines) in Figure 7 it was observed that there is a lower value of the peaks for the quench cooled samples than for the milled samples indicating a higher degree of disorder in the quench cooled samples. Such information was not obtainable from the Raman spectra and the XRPD diffractograms. PDF analysis of XRPD diffractograms is, thus, shown to provide additional information about the degree of disorder within the amorphous state. 

**Figure 8:**
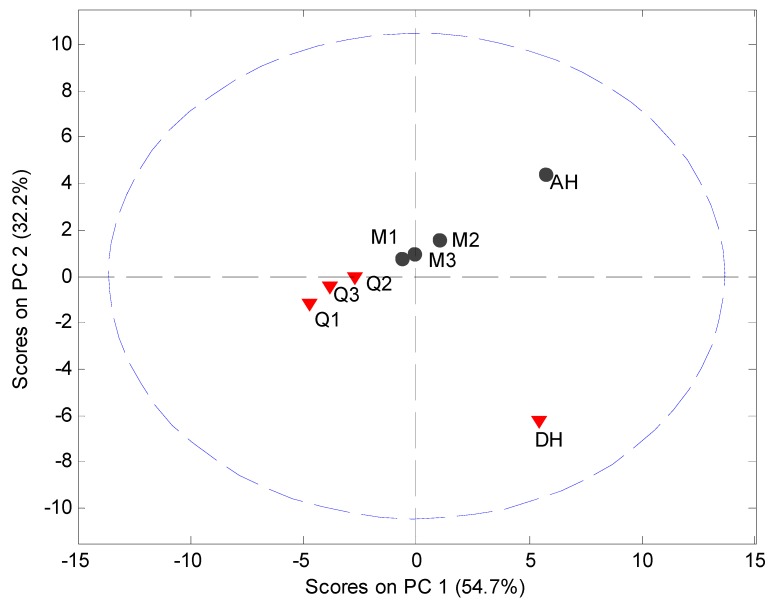
PCA score plot of PDF data (4 Å to 40 Å) using the 5° to 40° 2θ XRPD diffraction range. Milled samples (M1, M2 and M3), quench cooled samples (Q1, Q2 and Q3), crystalline anhydrate (AH) and crystalline dihydrate (DH).

MVDA, using SNV correction and mean centering, was carried out on the PDF data in the interval 4 Å to 40 Å to assess the structural disorder induced by milling or quench cooling. The PCA model utilized 2 PCs to explain 87.0% of the variation (Figure 8). It was observed that the crystalline samples were separated in the score plot from the processed samples and that the milled and quench cooled samples could be separated from each other. 

**Figure 9:**
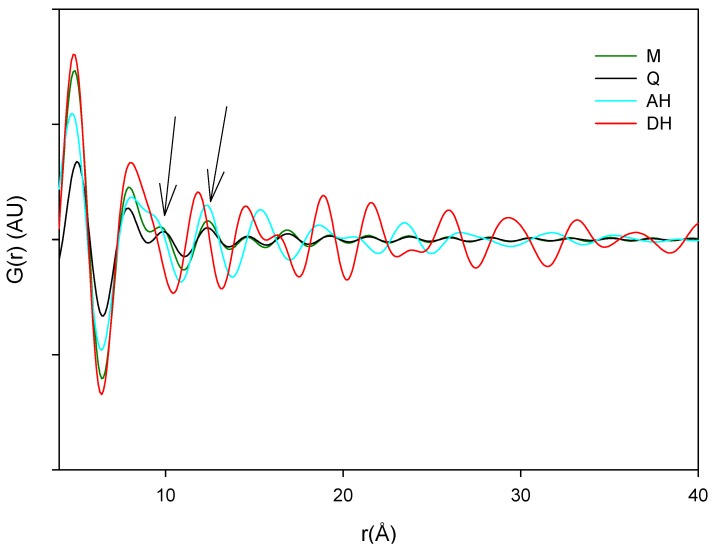
Averaged raw PDF traces of crystalline anhydrate (AH), crystalline dihydrate (DH), milled (M) and quench cooled (Q) samples.

The main source of variation in this PDF data set (indicated by separation in the direction of PC1) originates from disorder and hence lowered peak values. PC 2 is separating the crystalline starting materials and it was observed that the milled and quench cooled samples form a line with the crystalline AH sample. Furthermore, when the raw PDF data is inspected it can be observed that the milled and quench cooled samples seem to have a more pronounced similarity to the AH form than to the DH form (Figure 9). This is especially apparent from the first four peak positions of the processed samples that all coincide with peak positions in the PDF trace of the AH form and only with two of these four peak positions of the DH form (Figure 9B, black arrows). However, such correlations in PFD data between amorphous and crystalline forms have been shown to be influenced by the utilized radiation source. The use of a Cu anode was deemed to have to low a resolution for resolving structural similarities between two amorphous APIs and their respective crystalline counterparts [10]. It can therefore suggested that such findings would have to be reexamined utilizing a higher range in the 2θ diffraction angle or preferably with a higher energy radiation source.

**Figure 10:**
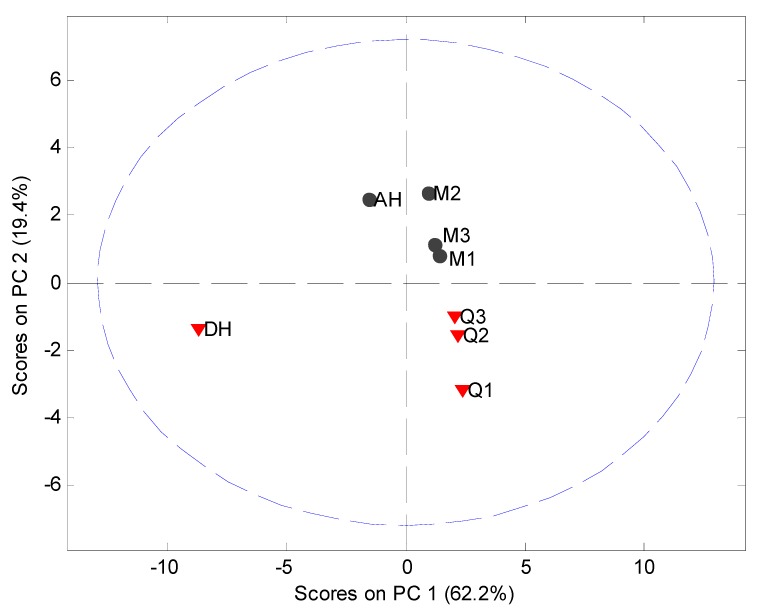
PCA score plot of PDF data (4 Å to 40 Å) using the 5° to 30° 2θ XRPD diffraction range. Milled samples (M1, M2 and M3), quench cooled samples (Q1, Q2 and Q3), crystalline anhydrate (AH) and crystalline dihydrate (DH).

The low energy Cu anodes capability to separate the PDF traces of the quench cooled and milled samples into two distinct groups was further investigated. PDF analysis was performed on a limited XRPD diffraction interval from 5° to 30° 2θ as opposed to the previous 5° to 40° 2θ interval. It is apparent that the PCA score plot of the PDF data is still capable of separating the differently prepared amorphous samples into two groups (Figure 10). It may subsequently be inferred that there must be a systematic difference between quench cooled and milled samples.

## 4. Conclusions

It was shown that atomic pair-wise distribution function (PDF) analysis provided a possibility to discriminate differently prepared disordered samples (milled samples, quench cooled samples) and starting crystalline materials, whereas X-ray powder diffraction without PDF analysis or Raman spectroscopy were not capable of separating these two preparation routes. PDF analysis suggested that quench cooled samples of the dihydrate form possessed a higher degree of disorder than the milled samples of the anhydrate form. PDF analysis utilizing a high energy radiation source may in future be utilized to obtain a deeper understanding of the disordered state and assessment of the inter-atomic distances within such a state.
